# Building Extraction from High–Resolution Remote Sensing Images by Adaptive Morphological Attribute Profile under Object Boundary Constraint

**DOI:** 10.3390/s19173737

**Published:** 2019-08-29

**Authors:** Chao Wang, Yi Shen, Hui Liu, Kaiguang Zhao, Hongyan Xing, Xing Qiu

**Affiliations:** 1 Collaborative Innovation Center on Forecast and Evaluation of Meteorological Disasters, Nanjing University of Information Science and Technology, Nanjing 210044, China; 2 College of Food, Agricultural, and Environmental Sciences, The Ohio State University, Wooster, OH 44691, USA; 3 College of Computer and Information Engineering, Hohai University, Nanjing 211100, China

**Keywords:** automatic building extraction, high–resolution, remote sensing, MAPs, AMAP–OBC

## Abstract

A novel adaptive morphological attribute profile under object boundary constraint (AMAP–OBC) method is proposed in this study for automatic building extraction from high-resolution remote sensing (HRRS) images. By investigating the associated attributes in morphological attribute profiles (MAPs), the proposed method establishes corresponding relationships between AMAP–OBC and building characteristics in HRRS images. In the preprocessing step, the candidate object set is extracted by a group of rules for screening of non-building objects. Second, based on the proposed adaptive scale parameter extraction and object boundary constraint strategies, AMAP–OBC is conducted to obtain the initial building set. Finally, a further identification strategy with adaptive threshold combination is proposed to obtain the final building extraction results. Through experiments of multiple groups of HRRS images from different sensors, the proposed method shows outstanding performance in terms of automatic building extraction from diverse geographic objects in urban scenes.

## 1. Introduction

With the continuous improvement of satellite and sensor technology, high–resolution remote sensing (HRRS) images have been widely used in many fields, such as updating geographic databases, creating urban thematic maps, etc. As buildings are among the most representative types of artificial targets in urban scenes, extraction of buildings from HRRS images is important in these applications [1,2,3]. Compared with traditional medium- and low-resolution remote sensing images, a great amount of semantic, textural, and spatial information of land covers is contained in HRRS images. Hence, HRRS images are appropriate data sources for building feature extraction. However, the increasing resolution of remote sensing images leads to the prominent phenomena of high intraclass variance and low interclass variance, which reduce the ability to distinguish buildings and other geographic objects [4].

In order to address this challenge, much effort has been made on importing spatial information as a supplement to spectral and textural features [5]. It has been proven that such information is highly effective in improving the ability to identify buildings in HRRS images [6,7]. In current works, machine learning-based methods are the main strategy for building a feature extraction [8,9,10,11]. However, such methods deeply rely on a huge number of samples and the effective selection of training samples. This means that in building feature extraction applications, such methods may not be implemented or obtain reliable results due to the lack of samples in HRRS images [4]. Meanwhile, more automatic building extraction methods with different strategies have been proposed, such as automatic building extraction with rooftop detectors [12], automatic building outline detection combined with geometric and spectral features [13], and the use of auxiliary data including light detection and ranging (LIDAR) [14] and terrestrial laser scanning (TLS) [15], etc. In addition, some building and non–building indices, such as the morphological building index (MBI) [16], shadow index [17], and vegetation index [18], have been widely used.

In recent years, building extraction with morphological attribute profiles (MAPs) has been proposed for HRRS images. As one of the most effective methods to model spatial and contextual information for the analysis of HRRS images, the operators in MAPs can be efficiently implemented based on the multiscale representation of land covers via tree structures [19]. Researchers have indicated that a combination of suitable scale parameters and morphological attributes can significantly improve the divisibility between buildings and other geographic objects [20,21]. However, there are still some restrictions in automatically extracting buildings from HRRS images by MAPs, as follows: (1) A reasonable set of scale parameters needs to be adaptively constructed. To extract buildings with different morphological attributes, it is crucial to produce a corresponding sequence of profiles by different scale parameters for each attribute. However, the theory of MAPs does not give explicit criteria about this and the scale parameters are mainly determined according to the experience of manual setting. (2) The connected area does not correspond to a geographic object. As the elementary unit of attribute extraction, the connected area for each pixel may invade into multiple geographic objects because it is determined only by the similarity of specific attributes between adjacent pixels. Therefore, it is hard to guarantee that the extracted result will accurately reflect the real attributes of the corresponding geographic object the current pixel belongs to. (3) For pixel-level results of MAPs and geographic objects, how to automatically acquire the final object-level building extraction results is also a challenging issue.

Concerning the above restrictions, a high-resolution remote sensing image building extraction method by AMAP–OBC is proposed, and the contributions of this study can be summarized as follows:

(1) A novel AMAP–OBC for automatic building extraction is proposed. By establishing the corresponding relationships between AMAP–OBC and characteristics of buildings in HRRS images, the set of scale parameters can be adaptively obtained, and the connected area for attribute extraction is restricted by the inherent boundaries of real geographic objects, which is beneficial for extracting more accurate attributes.

(2) In addition, a further identification strategy with adaptive threshold combination is proposed. It can break the semantic gap between the extracted building pixels and segmented geographic objects, and realize further screening of non–building objects with building pixels in the final results. 

This study mainly includes six sections: Section 2 contains the analysis of building characteristics in high-resolution remote sensing images; in Section 3, we briefly describe the MAP theory and constitution of the building attribute set; in Section 4, we elaborate on the implementation steps of the proposed method; Section 5 contains an analysis and discussion of the experiments; and in Section 6, we give the conclusion.

## 2. Analysis of Building Characteristics in HRRS Images

**Figure 1:**
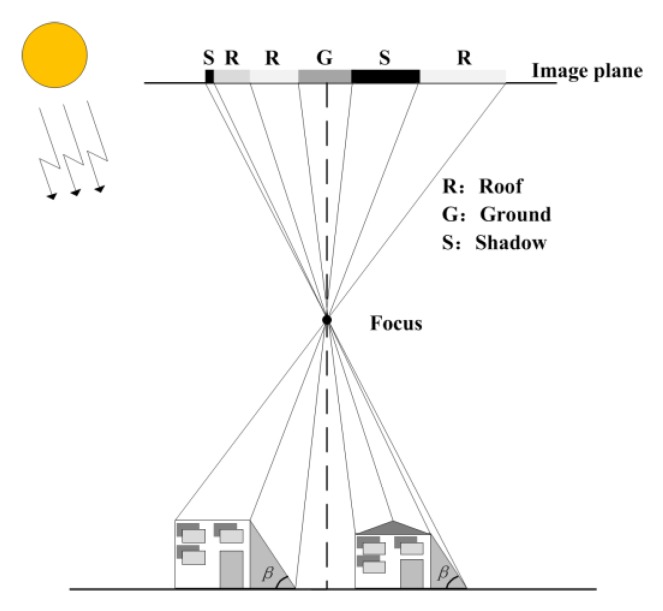
Geometric relationship between sensor, ground, and buildings.

The geometric relationship between the sensor, the ground, and buildings in remote sensing images is shown in Figure 1.

Roof, ground, and shadow, respectively, represent the roof of a building, the adjacent ground, and shadow caused by the building occluding sunlight. In general, different building roofs have different spectra and reflectivity due to material differences, so there may be significant differences in spectral and textural characteristics. However, since the pixels belonging to the roof of the same building have strong spectral and textural consistency, they are manifested as a homogeneous connected area constrained by the boundary of the building. In terms of geometric features, buildings usually behave as various rectangles or other regular shapes, and morphological attributes such as area, etc., are significantly different from other geographic objects such as roads and vehicles. The shadow of a building shows a significant dark color and a shape-regular connected area, and is distributed adjacent to the building, so it frequently produces confusion in the building extraction.

## 3. MAP Theory and Constitution of Building Attribute Set

### 3.1. MAP Theory

MAP theory is developed from set theory, in which adjacent pixels are first selected through spectral similarity and spatial connectivity to conduct the connected area, and then different operators are designed according to the characteristics of the geographic objects with different scale parameters and different attributes, and finally the extraction of specific objects is realized through differential processing [22]. Let M denote grayscale image, i denote a pixel point of the image, and k denote an arbitrary gray level. Then, a binary image $Th_{k}^{i}(M)$ can be obtained:

(1) $$Th_{k}^{i}(M)=\left\{ \begin{array}{l} 1,\;M\;(i)>k\\ 0,\;\mathrm{otherwise} \end{array}\right..$$

Traverse all pixels in an image to get a series $Th_{k}^{}(M)$ and set the maximum grayscale that satisfies the attribute constraint as the result of the attribute opening operation of point i:

(2) $$\Gamma^{\;i}(M)=\max\{k:i\in \Gamma^{\;i}(Th_{k}(M))\}.$$

By using the symmetry of attribute transformation, the attribute closed transformation Φ^*i*^(*M*) of point *i* can be obtained:

(3) $$\Phi^{\;i}(M)=\min\{k:i\in \Phi^{\;i}(Th_{k}(M))\},$$

where $\Phi^{i}(Th_{k}(M))=(\Gamma^{i}((Th_{k}(M){)}^{c}){)}^{c}$ denotes the attribute closed transformation of $Th_{k}^{}(M)$, and ${(Th_{k}^{}(M))}^{c}$ denotes the complementary set of $Th_{k}^{}(M)$. All pixels are traversed to obtain the attribute open transformation Γ(M) and the attribute closed transformation Φ(M) of M. On this basis, let $T=\{T_{0},T_{1},\ldots ,T_{W}\}$ denote the scale parameter set of MAPs and $T_{w}\in T$ denote the *w*th scale parameter; the difference between the adjacent scales of the attribute opening operation and closed operation result is taken separately, and the difference result constitutes the different morphological profile (DAP) transformation ΔΨ(M) of M, represented as follows:

(4) $$\Delta \Psi (M)=\{\Delta_{v}\Psi (M)\langle \begin{array}{l} \Phi_{T_{w}}(M)-\Phi_{T_{w-1}}(M),w=(W-v+1),\forall v\in [1,\cdot \cdot ,W]\\ \Gamma_{T_{w-1}}(M)-\Gamma_{T_{w}}(M),w=(v-W),\forall v\in [W+1,\cdot \cdot ,2W] \end{array}\rangle ,$$

where $\Gamma_{T_{w}}^{}(M)$ and $\Phi_{T_{w}}^{}(M)$ denote the attribute opening and closed transformation results obtained by scale $T_{w}$, respectively. Due to the difference between attributes, objects will have the greatest response on different scale parameters, then a set of pixels that conform to the attribute range of the building can be extracted according to this principle.

### 3.2. Constitution of Building Attribute Set

The constitution of the building attribute set is determined based on prior knowledge and the semantic characteristics contained in different attributes. According to the characteristics of the building analyzed in Section 2, this study constructed a building attribute set with four attributes: Area, diagonal, standard deviation, and normalized moment of inertia (NMI).

Among them, area reflects the size of the building; diagonal describes the diagonal length of the minimum external rectangle, thus reflecting the aspect ratio of the building; standard deviation describes the degree of gray variation inside the building; and NMI reflects the shape and gravity position of the building.

## 4. Method

**Figure 2:**
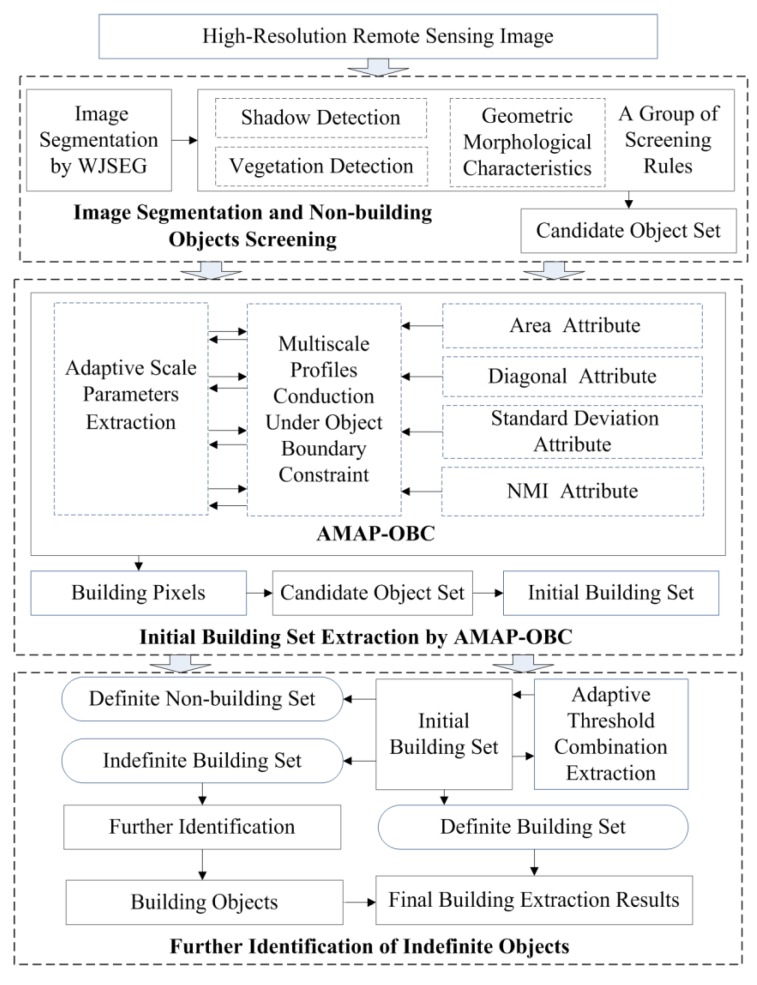
Flowchart of the proposed method. WJSEG, wavelet-JSEG; NMI, normalized moment of inertia; AMAP–OBC, adaptive morphological attribute profile under object boundary constraint.

The implementation of the proposed method mainly included image segmentation and non-building object screening, initial building set extraction by AMAP–OBC, and further identification of indefinite objects. A specific description of the implementation process is shown in Figure 2.

### 4.1. Image Segmentation and Non-Building Object Screening

#### 4.1.1. Image Segmentation by WJSEG

As shown in Figure 2, the discrete pixels in an HRRS image are first classified into geographic objects with semantic information through image segmentation, thus providing basic analysis units for building extraction [23]. The quality of segmentation has a strong influence on the practical value of the building extraction results [24]. Therefore, wavelet-JSEG (WJSEG), an effective high-resolution remote sensing image segmentation method, was adopted in this study [25].

Compared with the famous eCognition commercial software, WJSEG locates object boundaries more accurately in the complex background of a city, and helps to increase the transparency of the proposed method [26]. As an advanced multiscale segmentation method, WJSEG mainly includes four steps: Multiband image fusion, seed region conduction and secondary extraction, inter scale constraint segmentation, and region merging. The specific implementation steps can be found in [25].

#### 4.1.2. Non-Building Object Screening

On the basis of segmentation results, objects that differed significantly from the morphological characteristics of the building were removed, along with shadow and vegetation detection results. For each extracted object, the specific screening rules were as follows:

Rule 1: In order to reduce false positives caused by shadow, a pixel-level shadow detection method based on the Gaussian distribution background model theory was adopted. The specific implementation steps can be found in [27]. If the proportion of shadow pixels in an object was greater than 80%, the object was considered to be seriously affected by shadow and should be removed.

Rule 2: In order to reduce false positives caused by vegetation such as lawn and tree canopy, a vegetation index based on the red-green-blue (RGB) model was adopted to extract vegetation pixels. The specific implementation steps can be found in [18]. If the proportion of vegetation pixels in an object is greater than 80%, remove this object.

Rule 3: If there were fewer than 10 pixels in an object, the object was considered to be a dim or small target, such as a vehicle or noise, and it should be removed.

Rule 4: If the rectangular degree of an object was less than 0.8 and the length–width ratio of its minimum bounding rectangle was greater than 5, the object was considered to be a narrow target, such as a road or waterway, and it should be removed [28].

After the discrimination of all objects in segmentation results with the above group of rules, the remaining objects constituted a candidate object set as the input for subsequent building extraction.

### 4.2. Initial Building Set Extraction by AMAP–OBC

#### 4.2.1. Producing Attribute Profile Under the Object Boundary Constraint

**Figure 3:**
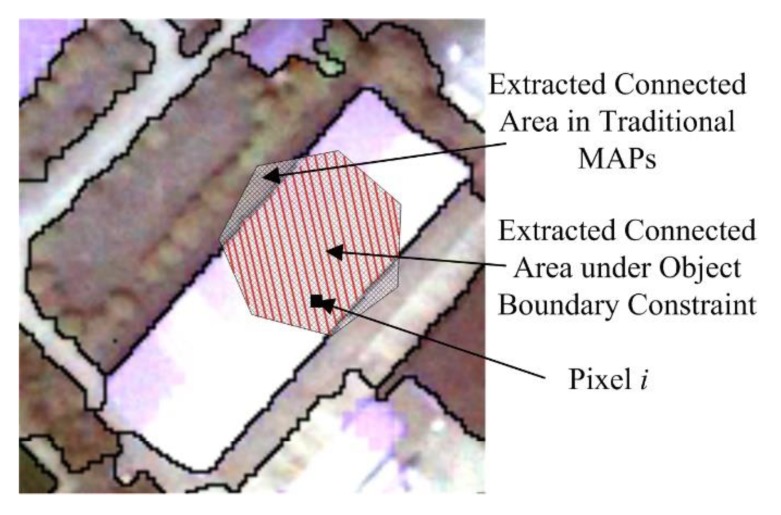
Extracted connected area.

During the process of calculating the attributes, the connected area for each pixel is produced by the similarity between adjacent pixels in traditional MAPs, as shown in Figure 3.

As shown in Figure 3, *i* represents a general pixel that belongs to an object in the candidate object set. The extracted corresponding connected area in a traditional MAP is expressed as the area with a black mesh pattern. It is shown that this area has invaded into adjacent objects. In this case, the inherent attributes of the current object cannot be accurately extracted. Therefore, this study retained only the pixels that were inside the object to produce the connected area for pixel *i*, as shown by the area with red lines. That is, the connected area would be constrained by the inherent boundary of the object pixel *i* belonging to, thus providing more accurate attributes for subsequent building extraction.

#### 4.2.2. Adaptive Scale Parameter Extraction

Based on the connected areas, the MAPs of different attributes were constructed according to Equations (1)–(4) in Section 3.1. In this process, whether the selection of the scale parameter set was reasonable was the key factor that affected the building extraction, which depended on the following: In urban scenes, building clusters in the same local area (such as a residential or industrial area) usually have a class of typical morphological attributes different from other features. Therefore, in the multiscale MAP of each attribute, it should be ensured that building clusters with typical attributes in the scene could be extracted through subsequent differential processing, while other objects were just removed. Based on this principle, this study proposed an adaptive extraction strategy for scale parameters, and the specific steps were as follows:

Step 1: Set the range and subintervals of the attribute interval to adaptively search the optimal scale parameters. According to suggestions regarding the fluctuation range of building attributes in [29,30,31], set area interval as [500, 28000], diagonal interval as [10, 100], standard deviation interval as [10, 70], and NMI interval as [0.2, 0.5], and divided each interval equally into 50 subintervals.

Step 2: For each attribute, let $SI_{x}^{}$ denote the *x*th subinterval. Under the object boundary constraint, the number of connected areas that met the requirements of the attribute range corresponding to $SI_{x}^{}$ was calculated, denoted by $Q_{x}$.

Step 3: Denote μ as an index of change degree. If it satisfies:

(5) $$(Q_{x}-Q_{x-1})>(Q_{x}+Q_{x-1})\times \mu .$$

The initial value of $SI_{x-1}^{}$ and the final value of $SI_{x}^{}$ are included as the optimal scale parameters. If it satisfies:

(6) $$(Q_{x}-Q_{x+1})>(Q_{x}+Q_{x+1})\times \mu .$$

The initial value of $SI_{x}^{}$ and the final value of $SI_{x+1}^{}$ are included as the optimal scale parameters; otherwise, continue the discrimination in the next interval. According to the ideal results of multiple experiments, it is suggested to set μ as 0.4 in this study.

The proposed adaptive scale parameter extraction strategy was based on the following corresponding relationships between morphological attributes and characteristics of buildings in HRRS images: If the number of connected areas satisfying the attribute range corresponding to $SI_{x}^{}$ was significantly higher than that of $SI_{x-1}^{}$, or when the number of connected areas satisfying the attribute range corresponding to $SI_{x+1}^{}$ was significantly lower than that of $SI_{x}^{}$, $SI_{x}^{}$ matched the typical morphological attributes of the building clusters that might exist in the scene. Therefore, it was necessary to consider $SI_{x}^{}$ as a typical interval, and the corresponding scale parameters need to be retained to ensure that the connected areas corresponding to $SI_{x}^{}$ could be effectively extracted during the differential processing.

Step 4: Traverse all intervals and use all optimized scale parameters extracted to form the final scale parameter set $T_{\mathrm{opt}}=\{T_{0},T_{1},\ldots ,T_{W}\}$. Then, the proposed AMAP–OBC could be produced based on $T_{\mathrm{opt}}$ and under object boundary constraint.

Step 5: Conduct DAP by the steps introduced in Section 3.1. On this basis, the pixels in each DAP that conformed to the attribute range of the building constituted a union set, and the pixels belonging to shadow and vegetation were removed. Finally, combined with the obtained set of candidate objects, all objects containing building pixels were retained to form the initial building set.

### 4.3. Further Identification of Indefinite Objects

The extraction results of the initial building set are not reliable, because the objects only need to meet the conditions for the existence of building pixels from AMAP–OBC. For this reason, this study partitioned the initial building set into a definite building set, an indefinite object set, and a definite non-building set, and further identified the indefinite objects. The specific steps were as follows:

Step 1: In the initial building set, let g denote the building pixel proportion in an object and $g_{\max}^{}$ denote the maximum of g, $g_{\mathrm{mid}}^{}=0.5\times g_{\max}$.

**Figure 4:**
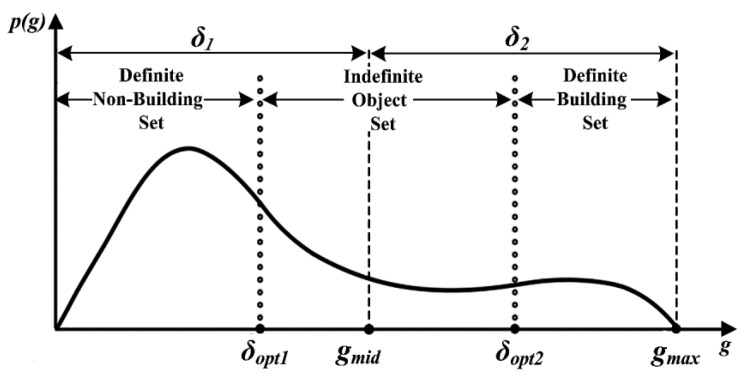
Further identification of initial building set.

As shown in Figure 4, p(g) represents the number of objects with g in the initial building set, and the fluctuation intervals of dynamic thresholds $\delta_{1}$ and $\delta_{2}$ are $\left(0,g_{\mathrm{mid}}^{}\right)$ and $\left(g_{\mathrm{mid}}^{},g_{\max}^{}\right)$, respectively. 

Step 2: Calculate the Jeffries Matusita (J–M) distance between any two objects that satisfy $g\in (0,\delta_{1}^{})$ and $g\in (\delta_{1}^{},g_{\mathrm{mid}}^{})$ to obtain the sum of these distances, $JM_{\delta_{1}}$. Similarly, $JM_{\delta_{2}}$ can be calculated based on the objects that satisfy $g\in (g_{\mathrm{mid}},\delta_{2}^{})$ and $g\in (g_{2},g_{\max})$. Let $JM_{\delta_{1,2}}=JM_{\delta_{1}}+JM_{\delta_{2}}$; by traversing all possible combinations of $\delta_{1}$ and $\delta_{2}$, the optimal combination can be adaptively extracted when the minimum value of $JM_{\delta_{1,2}}$ is obtained, as shown by $\delta_{\mathrm{opt}1}$ and $\delta_{\mathrm{opt}2}$ in Figure 4. On this basis, the definite building set, indefinite object set, and definite non-building set are extracted.

Step 3: For each object $R_{\mathrm{indefinite}}^{}$ in the indefinite object set, further identification was made. Let the sum of J–M distances between $R_{\mathrm{indefinite}}^{}$ and all objects in the definite building set be $JM_{\mathrm{true}}$, and the sum of J–M distances between $R_{\mathrm{indefinite}}^{}$ and all objects in the definite non-building set be $JM_{false}$. If $JM_{true}<JM_{false}$, put $R_{\mathrm{indefinite}}^{}$ in the definite building set; otherwise, put $R_{\mathrm{indefinite}}^{}$ in the definite non-building set. 

Step 4: Traverse all objects in the indefinite object set to obtain the final building extraction results.

## 5. Experiments and Discussion

In the experiments, three datasets of HRRS images were used. Combining statistical accuracy and visual inspection, the performance of the method in this study was verified by comparison with a variety of advanced building extraction methods.

### 5.1. Datasets and Experimental Strategy 

#### 5.1.1. Dataset Description

**Figure 5:**
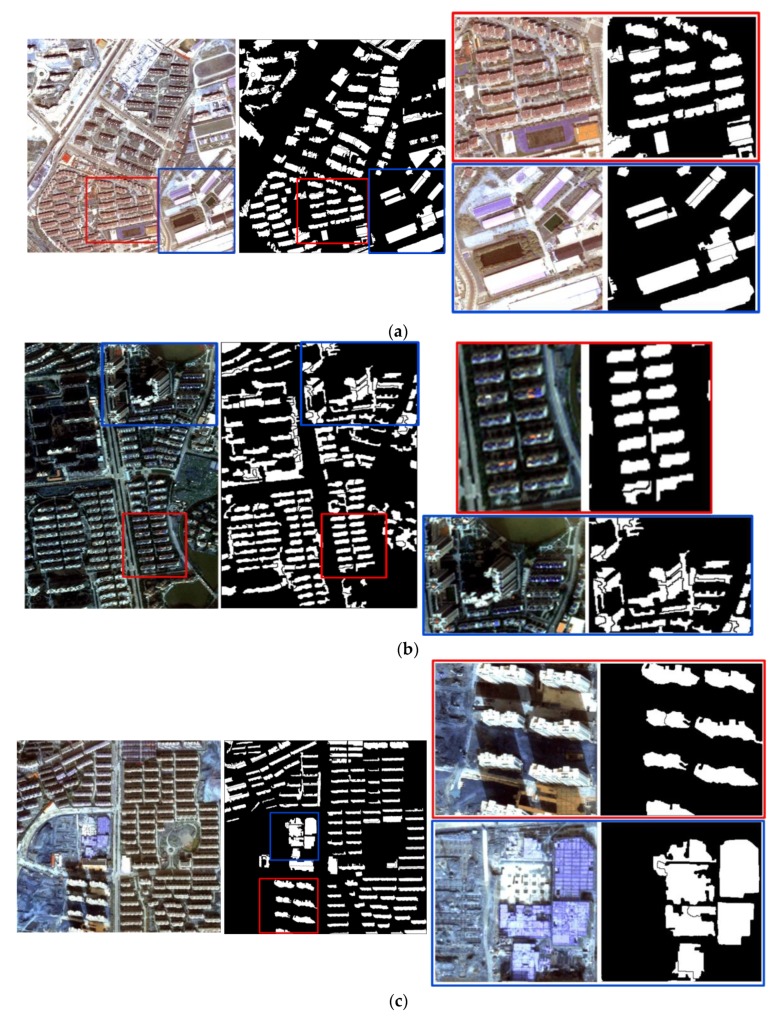
Three datasets and corresponding ground truth maps: (**a**) Dataset 1 and patches I1 (red box) and I2 (blue box); (**b**) dataset 2 and patches I3 (red box) and I4 (blue box); and (**c**) dataset 3 and patches I5 (red box) and I6 (blue box).

Dataset 1 was a pan-sharpened WorldView image with red, green, and blue bands of Chongqing, China; the acquisition date was August 2011, the spatial resolution was 0.5 m, and the size was 1370 pixels × 1370 pixels, as shown in Figure 5a. Dataset 2 was an aerial remote sensing image with red, green, and blue bands of Nanjing, China; the acquisition date was October 2011, the spatial resolution was 2 m, and the image size was 300 pixels × 500 pixels, as shown in Figure 5b. Dataset 3 was a WorldView pan-sharpened image with red, green, and blue bands of Nanjing, China; the acquisition date was December 2012, the spatial resolution was 0.5 m, and the image size was 1400 pixels × 1400 pixels, as shown in Figure 5c. In addition, the ground truth maps were manually delineated by field investigation and visual interpretation, in which white objects represent buildings and black objects represent non-buildings. Some representative areas marked in red boxes (patches I1, I3, and I5) and blue boxes (patches I2, I4, and I6) in Figure 5 were chosen for detailed comparison and analysis.

The reasons for selecting these three datasets for the experiments were as follows: (1) Airborne and satellite-borne sensors are currently the two principal forms of HRRS image acquisition. Using these datasets was helpful to analyze the applicability of the proposed method for different data sources. (2) These datasets were typical urban scenes, mainly composed of land covers such as buildings, roads, vegetation, wasteland, shadows, etc., which was helpful to verify the stability and reliability of the proposed method. (3) The acquisition seasons of these datasets were different, which was helpful to analyze the influence of vegetation factors on the extraction of buildings. (4) As an aerial remote sensing image, dataset 2 had a large oblique imaging angle. By comparing with the other two datasets, it was helpful to analyze the influence of building inclination, especially for high-rise buildings, on the proposed method.

#### 5.1.2. Experimental Setup

In order to analyze the performance of this method comprehensively and objectively, this study used four advanced building extraction methods for comparative experiments: The traditional MAP method (method 1) [5], the MBI-based method (method 2) [16]; the top-hat filter and k-means classification based method (method 3) [7], and the gray-level co-occurrence matrix (GLCM) and support vector machine (SVM) based method (method 4) [20]. By comparing with method 1, it was helpful to analyze the validity of the proposed boundary constraint strategy. Methods 2 and 3 were automatic building extraction methods: Building index and rooftop detector methods, respectively. Method 4 was the machine learning method. These three types of advanced methods were adopted to evaluate the overall performance of the proposed method. Methods 1 and 2 were pixel-based, and it was difficult to compare their building extraction effect directly with the object-based method. Therefore, based on the building pixels extracted from methods 1 and 2, the subsequent implementation steps were the same as the proposed method. At the same time, in order to ensure consistency of the basic units, the segmentation in methods 3 and 4 was replaced with WJSEG, and the other implementation steps and parameter settings were consistent with the original reference. The parameter setting of the proposed method and the corresponding basis were given in Section 4. On this basis, the adaptively extracted scale parameters were set, and the parameter combinations of $\delta_{opt1}$ and $\delta_{opt2}$ are shown in Table 1, Table 2, Table 3 and Table 4.

### 5.2. Experimental Results and Accuracy Evaluation

#### 5.2.1. General Results and Analysis of Datasets

**Figure 6:**
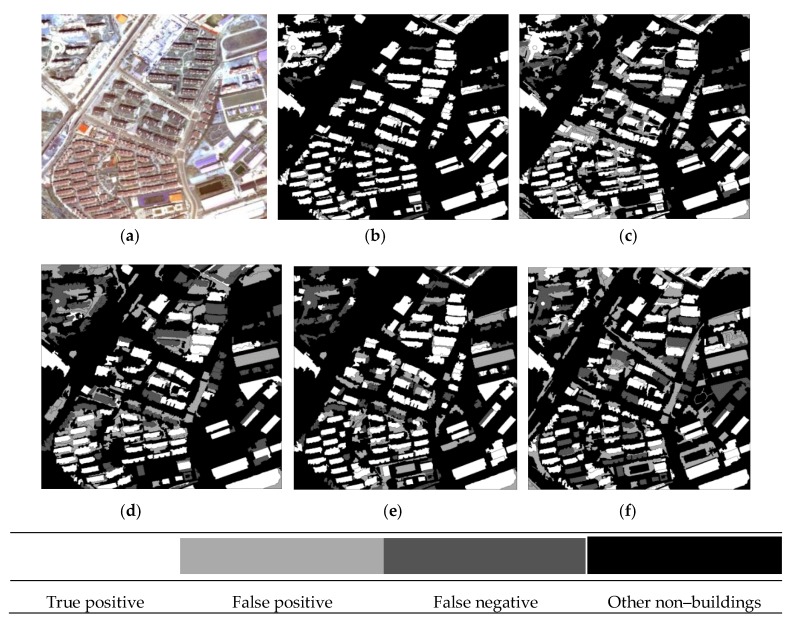
Building extraction results of dataset 1: (**a**) Original image; (**b**) proposed method; (**c**) method 1; (**d**) method 2; (**e**) method 3; and (**f**) method 4.

**Figure 7:**
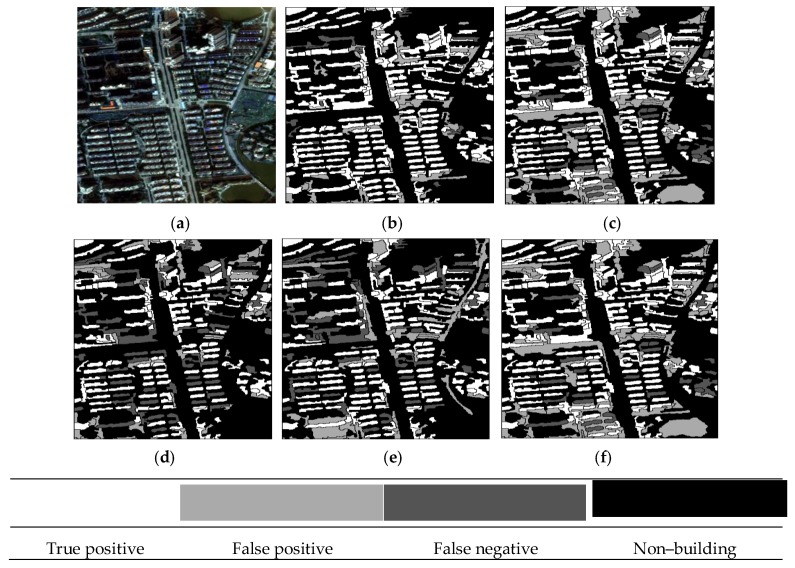
Building extraction results of dataset 2: (**a**) Original image; (**b**) proposed method; (**c**) method 1; (**d**) method 2; (**e**) method 3; and (**f**) method 4.

**Figure 8:**
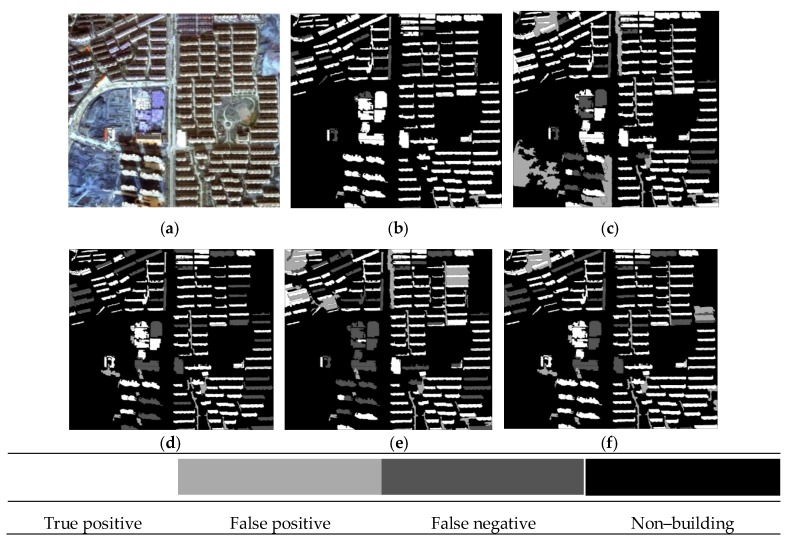
Building extraction results of dataset 3: (**a**) Original image; **(b**) proposed method; (**c**) method 1; (**d**) method 2; (**e**) method 3; and (**f**) method 4.

The building extraction results of the three datasets are given in Figure 6, Figure 7 and Figure 8, in which the true positive (TP), false positive (FP), false negative (FN), and other non–buildings are represented by four colors.

The quantitative results of the different methods are reported in Table 5, Table 6 and Table 7. By the statistical accuracy and visual inspection shown in the three groups of experiments, overall accuracy (OA) of the proposed method reached more than 90%, and the fluctuation range was less than 2%, which was significantly higher than the other four comparison methods. Therefore, among the challenges brought by the different data sources, the proposed method had high accuracy, high stability, and high reliability. Moreover, it also shows that the seasonal differences in the collection of the three datasets and the existing differences in building inclination did not significantly affect the extraction accuracy of the proposed method. 

Compared with the proposed method, the FPs of method 1 in the three groups of experiments were significantly reduced, and there was no significant difference of FNs between the two methods. This shows that MAPs had the advantage of being very sensitive to potential buildings in the image. On the other hand, it also shows that the traditional MAP strategy of constructing a connected area only based on similarities between adjacent pixels had difficulty accurately describing the inherent attributes of the object, which led to an increase in FPs and a significant decrease in OA. Therefore, the object boundary constraint strategy proposed in this study was feasible, effective, and necessary.

Except for the OA of method 3 in dataset 1 (82.4%), the OA of methods 2 and 3 in the three groups of experiments was lower than 80%. This was mainly due to the fixed-shape structural elements adopted by these methods in constructing the descriptors. These kinds of descriptors were only sensitive to the pixels that belong to buildings with similar morphological characteristics of structural elements, while ignoring the diversity of building shapes and sizes in urban scenes, so it was difficult to obtain ideal results. In addition, since shadows were not considered in method 3, there was a certain amount of fake shadow objects in the final building extraction results.

Since method 4 was a classification method based on machine learning, it had higher requirements for an abundance of samples. However, there were only 833, 462, and 212 samples after WJSEG segmentation in datasets 1, 2, and 3, respectively, so it was difficult to reflect the real accuracy that method 4 could reach. Therefore, although the OA of method 4 fluctuated slightly and exceeded 80% in the three groups of experiments, FPs and FNs show large fluctuations. In addition, as the OA in dataset 1 with more samples (83.2%) was higher than that in dataset 2 (80.1%) and dataset 3 (80.7%), we believed that with increased samples, the OA of method 4 would be significantly improved.

#### 5.2.2. Visual Comparison of Representative Patches

**Figure 9:**
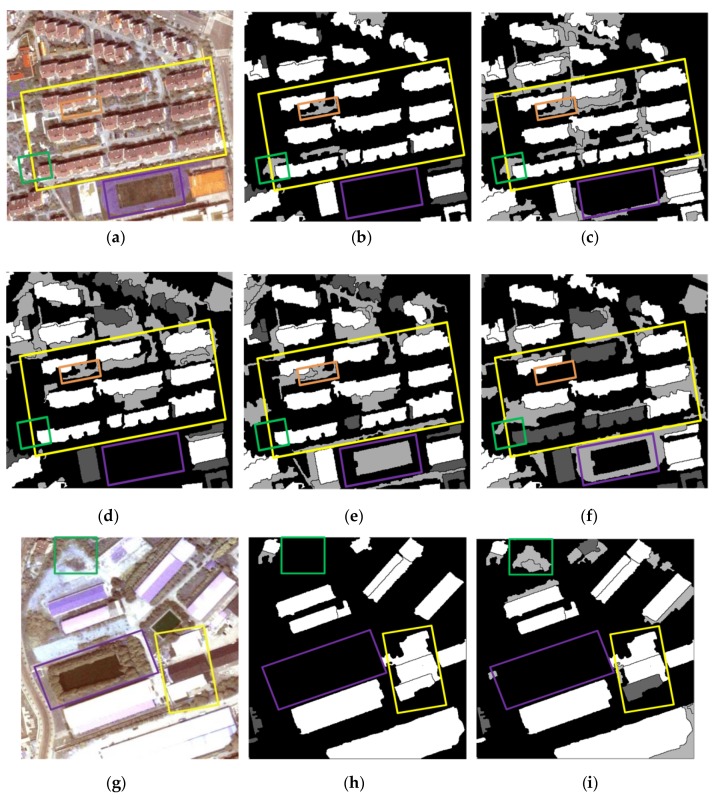

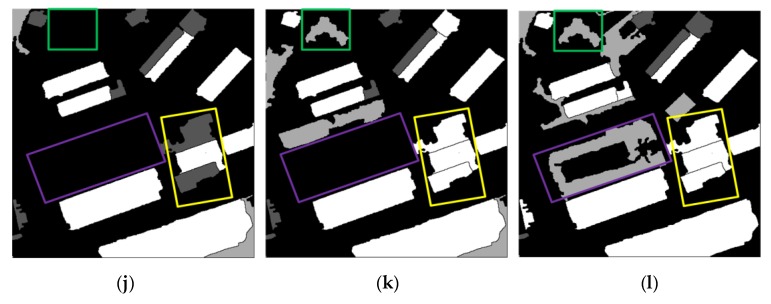
Building extraction results of patches I1 and I2: (**a**) Patch I1; (**b–f**) results obtained in patch I1 using the proposed method and methods 1, 2, 3, and 4 respectively; (**g**) patch I2; (**h–l**) results obtained in patch I2 using the proposed method and methods 1, 2, 3, and 4, respectively.

**Figure 10:**
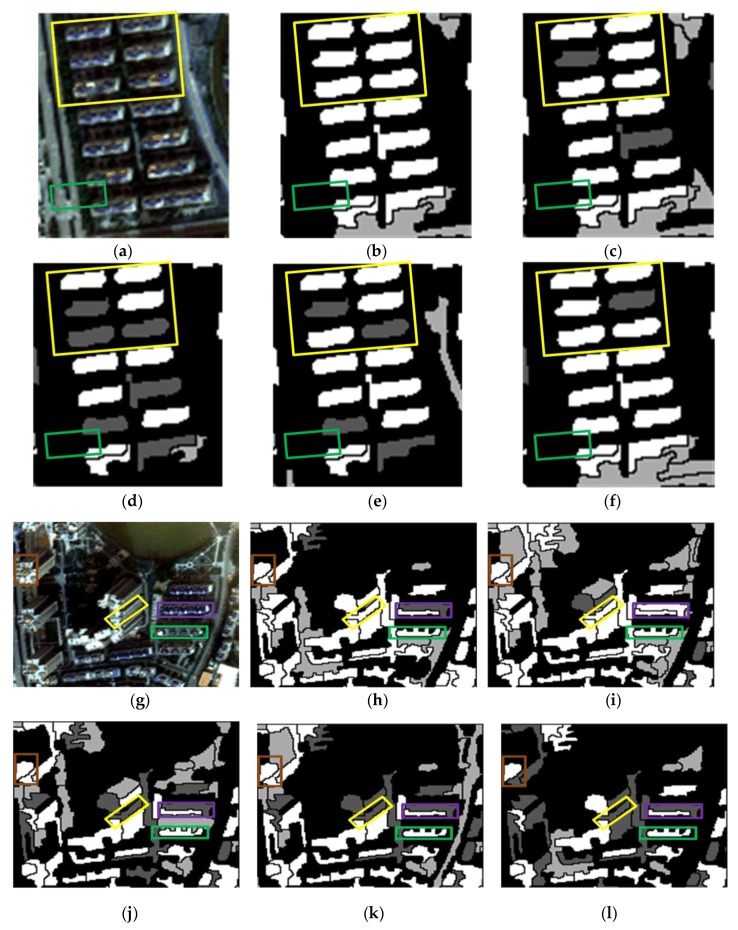
Building extraction results of patches I3 and I4: (**a**) Patch I3; (**b–f**) results obtained in patch I3 using the proposed method and methods 1, 2, 3, and 4, respectively; (**g**) patches; (**h–l**) results obtained in patch I4 using the proposed method and methods 1, 2, 3, and 4, respectively.

**Figure 11:**
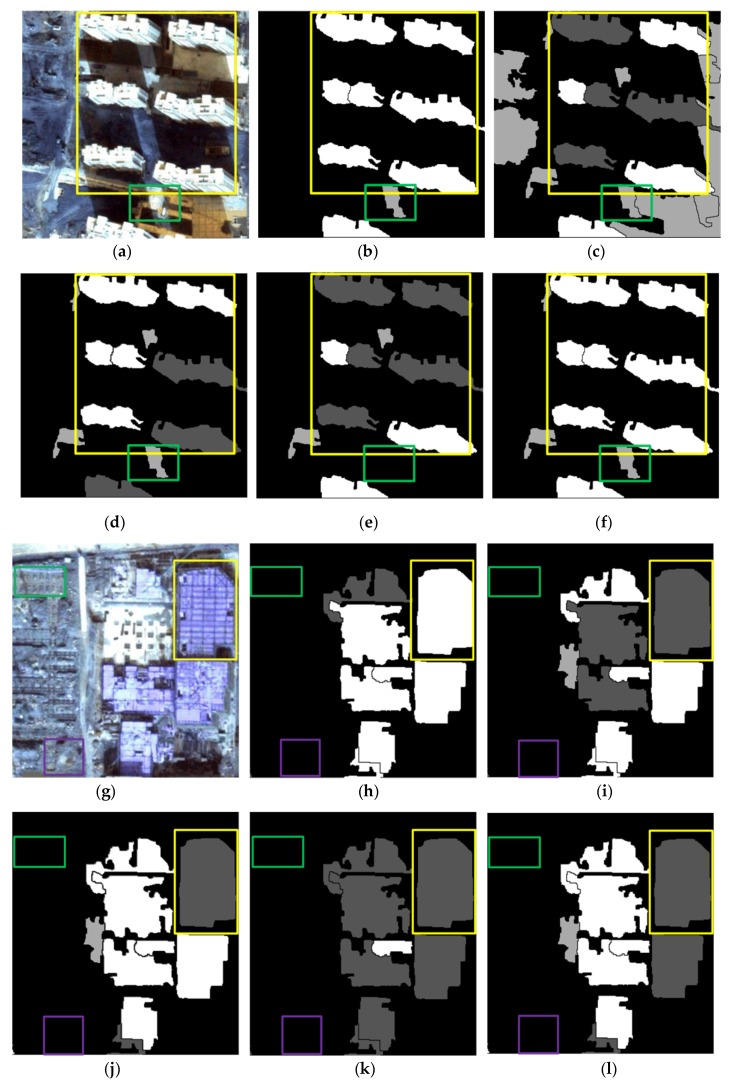
Building extraction results of patches I5 and I6: (**a**) Patch I5; (**b–f**) results obtained in patch I5 using the proposed method and methods 1, 2, 3, and 4, respectively; (**g**) patch I6; (**h–l**) results obtained in patch I6 using the proposed method and methods 1, 2, 3, and 4, respectively.

The results of the representative patches in each dataset are reported in Figure 9 (patches I1 and I2), Figure 10 (patches I3 and I4), and Figure 11 (patches I5 and I6). The results obtained by the proposed method were the most complete and precise in most scenes. The results for each representative patch were discussed as follows.

As the most common types of buildings in urban HRRS images, residential and industrial buildings are always regions of interest (ROIs) in related applications. Therefore, the following analysis and discussion were focused on the extraction effects of these two types of buildings. First of all, for residential building with small size (e.g., residential buildings in the yellow rectangle of I1) and industrial buildings with large size (e.g., industrial buildings in the yellow rectangle of I6), the analysis shows that the adopted WJSEG could accurately extract their complete contours with different shapes, thus providing effective analysis units for subsequent building extraction. In terms of residential building extraction, the proposed method accurately extracted the vast majority of buildings, as shown in the yellow rectangles of I1, I3, and I5, which was significantly better than the other comparison methods. At the same time, mixed shadows, vegetation, roads, and other artificial targets (e.g., green rectangle in I3) were effectively filtered out. Among the other four comparison methods, the extraction effect of method 4 was better than that of the other three. Especially in I5, due to the irregular shapes of the buildings, methods 1, 2, and 3 all had serious FPs and FNs. In the building extraction of industrial areas, for example, in the yellow rectangle of I2, only methods 3 and 4 and the proposed method completely extracted three buildings, but at the same time methods 3 and 4 erroneously detected the wasteland in the green rectangle of I2 as a building. As for common stacking areas of production materials in industrial areas (e.g., the green rectangle in I6) and wasteland around factory buildings (e.g., the purple rectangle in I6), all five methods could extract them correctly. In addition, geographic objects with similar morphological features of building, such as playground (e.g., the purple rectangle in I1) and pool (e.g., the purple rectangle in I2), which were located around the two types of buildings, were also effectively screened by the proposed method. To sum up, these representative patches show that the proposed method was significantly better than the other four comparison methods.

On this basis, we further discussed the influence of shadow, vegetation, and building inclination on the extraction effect of the proposed method. (1) In terms of shadow, the shadow detection strategy introduced in the proposed method already filtered out most shadow objects. However, there were a few ground surfaces (e.g., the green rectangle in I5) with similar textures and morphological features of buildings between adjacent shadows that were erroneously detected as buildings. (2) In terms of vegetation, although the collection seasons of the three datasets were summer, autumn, and winter, the vegetation index basically filtered out vegetation objects, such as canopies and lawns in the yellow and purple rectangles of I2. Obvious FNs only existed in areas where buildings and low canopies with weak edges were densely distributed (e.g., the green and brown rectangles in I1). (3) Since the building inclination effect was more prominent in high-rise buildings in aerial remote sensing images, we chose I4, belonging to dataset 2, for detailed discussion. Through analysis, we found that the building side elevation generated by the building inclination effect would result in two situations after segmentation: (1) When the side elevation and the roof were divided into the same object, such as yellow and green rectangles, these objects were correctly extracted. After visual inspection of all the datasets, it was also rare to find any FPs or FNs caused by this situation. (2) When the side elevation was regarded as an individual object in the segmentation results, FNs (e.g., the purple rectangle) or filtering out as shadow (e.g., the brown rectangle) might occur. In spite of this, we found that the roofs corresponding to these side elevations were accurately extracted, so it still had certain reference value in practical application.

### 5.3. Analysis of the Impact on the Overall Accuracy with Different μ

**Figure 12:**
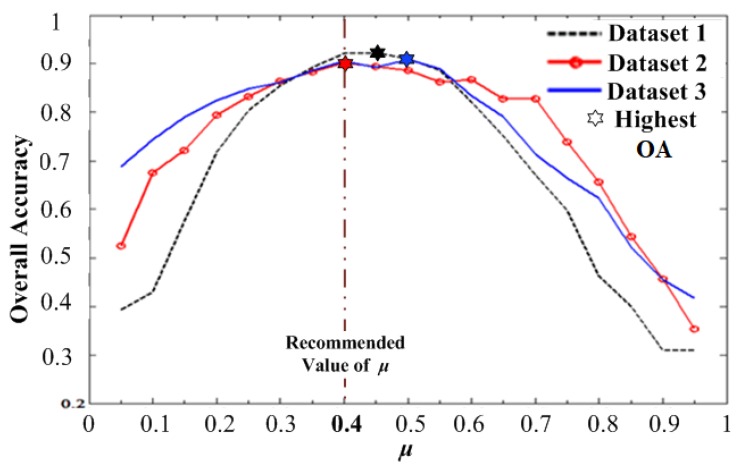
Impact on the overall accuracy by different μ.

During the adaptive scale parameter extraction process proposed in this study, the change degree index μ in Equations (5) and (6) was used to determine the degree of difference between extracted typical interval and adjacent intervals. In order to specify the setting basis of μ, the impact on OA with different μ was analyzed in this study. As shown in Figure 12, the horizontal coordinate was μ, the interval was 0.05, the longitudinal coordinate was OA, and the experimental results of three datasets were represented by curves in different styles.

As shown above, in the three dataset experiments, with the continuous increase of μ, OA shows a similar trend of gradually increasing at first and then rapidly decreasing after reaching the peak. Among them, μ=0.45, μ=0.4, and μ=0.5 corresponded to the peaks of the overall accuracy curves with 92.3%, 90.2%, and 90.8% in the experiments of datasets 1, 2, and 3, respectively. The detailed *μ*-OA values in the three groups of experiments are shown in Table 8.

Through analysis we found that when μ was set as 0.4, OA could reach 92.1% and 90.5%, and was only slightly lower, by 0.02% and 0.03%, than the corresponding highest OA in datasets 1 and 3, respectively. This means that the ideal results could be obtained in all three dataset experiments by setting μ as 0.4. Therefore, considering the requirements of automation and reliability, it is suggested to directly set μ as 0.4 in practical applications.

## 6. Conclusions

Aiming at the restrictions in automatically extracting buildings by MAPs, a novel adaptive morphological attribute profile under object boundary constraint (AMAP–OBC) was proposed in this study. By establishing the corresponding relationships between AMAP–OBC and characteristics of buildings in HRRS images, a set of scale parameters could be adaptively obtained, and meanwhile the connected area extraction was restricted by the inherent boundaries of geographic objects. On this basis, the final building extraction results were obtained by a further identification strategy with an adaptive threshold combination. In experiments with urban high-resolution remote sensing images, the proposed method was significantly better than four comparison methods in statistical accuracy and visual inspection, and OA reached more than 90%, while FPs and FNs were lower than 7% and 6%, respectively. Therefore, the proposed method showed outstanding performance in terms of building extraction from diverse objects in urban districts.

## Figures and Tables

**Table 1:** Extracted scale parameter set of dataset 1.

Attribute	Scale Parameter Set of Dataset 1
Area	(1050, 2149, 1600, 2699, 4900, 5999, 6000, 7099, 7650, 8749, 8750, 9849, 9300, 10,399, 10,400, 11,499, 15,350, 16,499, 17,000, 18,099, 22,500, 23,599)
Diagonal	(20.8, 24.3, 29.8, 33.3, 38.8, 42.3, 49.6, 53.1, 65.8, 69.3, 74.8, 78.3, 82, 85.5, 92.8, 96.3)
NMI	(0.2, 0211, 0.224, 0.235, 0.254, 0.265, 0.284, 0.295, 0.302, 0.313, 0.35, 0.361, 0.38, 0.391,0.427,0.439,0.482,0.493)
Standard deviation	(18, 19.9, 23, 24.9, 29, 30.9, 32, 33.9, 45, 46.9, 60, 61.9, 64, 65.9)

**Table 2:** Extracted scale parameter set of dataset 2.

Attribute	Scale Parameter Set of Dataset 2
Area	(500, 1599, 1600, 2699, 2700, 3799, 4350, 5449, 6000, 6549)
Diagonal	(10, 13.5, 15.4, 18.9, 20.8, 24.3, 28, 31.5, 35.2, 38.7, 46, 49.5, 55, 58.5, 64, 67.5, 69.4, 72.9)
NMI	(0.2, 0211, 0.212, 0.223, 0.23, 0.241, 0.26, 0.271, 0.314, 0.325, 0.35, 0.361, 0.368, 0.379, 0.416, 0.427)
Standard deviation	(10, 11.9, 15, 16.9, 24, 25.9, 28, 29.9, 34, 35.9, 40, 41.9, 46, 47.9, 50, 51.9, 54, 55.9)

**Table 3:** Extracted scale parameter set of dataset 3.

Attribute	Scale Parameter Set of Dataset 3
Area	(2150, 3249, 3250, 4349, 6000, 7099, 8200, 9299, 9850, 10,949, 12,600, 13,699, 15,900, 16,999, 19,200, 20,229)
Diagonal	(17.2, 20.7, 24.4, 27.9, 31.6, 35.1, 37, 40.5, 47.8, 51.3, 58.6, 62.1, 64, 67.5, 71.2, 74.7, 89.2, 92.7, 94.6, 98.1)
NMI	(0.2, 0.211, 0.224, 0.235, 0.242, 0.253, 0.296, 0.307, 0.332, 0.343, 0.35, 0.361, 0.38, 0.391, 0.47, 0.481)
Standard deviation	(12, 13.9, 16, 17.9, 20, 21.9, 22, 23.9, 25, 26.9, 30, 31.9, 33, 34.9, 38, 39.9, 40, 41.9, 46, 47.9, 50, 51.9, 56, 57.9, 62, 63.9)

**Table 4:** Extracted combinations of $\delta_{opt1}$ and $\delta_{opt2}$ in the three datasets.

Dynamic Threshold	Dataset 1	Dataset 2	Dataset 3
$\delta_{opt1}$	0.43	0.35	0.39
$\delta_{opt2}$	0.81	0.77	0.75

**Table 5:** Evaluation of building extraction accuracy in dataset 1. OA, overall accuracy; FP, false positive; FN, false negative.

Method/Indicator	OA (%)	FP (%)	FN (%)	Kappa
Evaluation Criteria	The Higher the Better	The Lower the Better	The Lower the Better	The Higher the Better
Proposed method	92.1	4.71	3.12	0.782
Method 1	72.9	22.6	4.43	0.556
Method 2	71.9	16.1	12.1	0.542
Method 3	83.1	6.83	9.82	0.644
Method 4	83.8	10.7	5.99	0.663

**Table 6:** Evaluation of building extraction accuracy in dataset 2.

Method/Indicator	OA (%)	FP (%)	FN (%)	Kappa
Evaluation Criteria	The Higher the Better	The Lower the Better	The Lower the Better	The Higher The Better
Proposed method	90.2	6.95	3.25	0.780
Method 1	76.9	17.0	6.06	0.543
Method 2	75.5	10.8	13.6	0.527
Method 3	78.7	8.89	12.6	0.568
Method 4	80.1	5.64	14.3	0.594

**Table 7:** Evaluation of building extraction accuracy in dataset 3.

Method/Indicator	OA (%)	FP (%)	FN (%)	Kappa
Evaluation Criteria	The Higher the Better	The Lower the Better	The Lower the Better	The Higher the Better
Proposed method	90.5	4.65	5.12	0.766
Method 1	78.6	13.9	7.44	0.529
Method 2	76.7	9.30	13.9	0.501
Method 3	72.6	12.6	14.9	0.456
Method 4	80.9	9.30	9.77	0.563

**Table 8:** Detailed *μ*-OA values in three dataset experiments.

**Dataset 1**	***μ***	0.05	0.1	0.15	0.2	0.25	0.3	0.35	0.4	0.45	0.5
	**OA (%)**	39.3	42.9	57.6	71.8	80.4	85.5	89.3	92.1	92.3	91.0
	***μ***	0.55	0.6	0.65	0.7	0.75	0.8	0.85	0.9	0.95	
	**OA (%)**	88.6	82.1	75.1	67.1	59.8	46.2	39.9	30.8	30.8	
**Dataset 2**	***μ***	0.05	0.1	0.15	0.2	0.25	0.3	0.35	0.4	0.45	0.5
	**OA (%)**	52.5	67.6	72.1	79.4	83.2	86.4	88.4	90.2	89.5	88.7
	***μ***	0.55	0.6	0.65	0.7	0.75	0.8	0.85	0.9	0.95	
	**OA (%)**	86.2	86.8	82.7	82.7	73.9	65.7	54.3	45.7	35.3	
**Dataset 3**	***μ***	0.05	0.1	0.15	0.2	0.25	0.3	0.35	0.4	0.45	0.5
	**OA (%)**	68.8	74.4	78.9	82.5	84.9	86.1	88.6	90.5	89.3	90.8
	***μ***	0.55	0.6	0.65	0.7	0.75	0.8	0.85	0.9	0.95	
	**OA (%)**	88.9	83.4	79.1	71.3	66.4	62.3	52.1	45.4	41.6	
